# Interactive workshop to develop implementation framework (i-PARIHS) resources to support practice facilitation

**DOI:** 10.1186/s43058-020-00046-0

**Published:** 2020-06-18

**Authors:** Sarah C. Hunter, Bo Kim, Alison L. Kitson

**Affiliations:** 1 College of Nursing and Health Sciences, Flinders University, Sturt Road, Bedford Park, Adelaide, South Australia 5042 Australia; 2 Caring Futures Institute, Flinders University, Sturt Road, Bedford Park, Adelaide, South Australia 5042 Australia; 3 Center for Healthcare Organization and Implementation Research, US Veterans Health Administration, 150 South Huntington Avenue, Boston, MA 02130 USA; 4 Department of Psychiatry, Harvard Medical School, 25 Shattuck Street, Boston, MA 02115 USA

**Keywords:** Practice facilitation, Implementation science, Knowledge translation, i-PARIHS framework

## Abstract

**Background:**

Practice facilitation is a method used to address the complexity associated with implementation of innovations into primary care. To provide support, we propose that the i-PARIHS (Integrated Promoting Action on Research Implementation in Health Services) framework could support practice facilitators. The i-PARIHS framework positions facilitation as a core element for successful implementation. Therefore, the aim of this study was to provide support to practice facilitators whilst simultaneously gaining feedback on what facilitators in practice need in regard to support with operationalising the i-PARIHS framework in practice.

**Methods:**

This study involved the delivery of a 1-h workshop on the i-PARIHS framework at the *2018 International Conference on Practice Facilitation*. The authors provided an overview of the i-PARIHS framework, how it can be used to support the facilitation of innovations into practice, and finally, attendees worked through facilitation scenarios and applied an i-PARIHS resource. At the end of the workshop, attendees were invited to participant in the research component, by completing a post-workshop survey on the workshop content and the i-PARIHS resource.

**Results:**

Participants were highly engaged and enthusiastic about the workshop. Participants reported that an introduction to implementation frameworks was valuable and the example of how the i-PARIHS framework had been used in a previous project was helpful. Overall, this study identifies how framework informed facilitation helped participants feel more equipped to conduct systematic facilitation and that the development of i-PARIHS resources would be helpful in their everyday work.

**Conclusions:**

This study demonstrates that the existence of implementation frameworks is not sufficient to provide support to those who facilitate in the real world. The current study introduced practice facilitators to the i-PARIHS framework, and the findings demonstrate the need to develop and refine existing i-PARIHS resources to support facilitation. Specifically, the next steps stemming from this study will be to (i) continue to utilize workshops for sharing and refining tools, (ii) allocate development efforts to tools that assist with planning, (iii) focus on tool provision mechanisms that keep user-friendliness in mind, and (iv) translate the i-PARIHS facilitation checklist from academic language into more practical and user-friendly language.

Contributions to the literature

- This study demonstrates the use of an interactive workshop as a feasible mechanism for introducing front-line practice facilitators to implementation science concepts and practical guidance that it can offer.
- The study findings indicate that practice facilitators are currently most interested in structured tools that can help them plan (more than guide or evaluate) their facilitation projects.
- This effort serves as a basis upon which to subsequently build a program of work that focuses on developing a suite of pragmatic resources that adapt the i-PARIHS framework for front-line use.

## Background

Successful implementation of new innovations and evidence into clinical practice can be very challenging and time-consuming [1]. Practice facilitation is a commonly used and practical method for supporting implementation in the context of primary care. Practice facilitation is a multifaceted approach involving skilled individuals who enable others through a multitude of activities (e.g. stakeholder engagement, provider feedback, and education) to address challenges associated with implementation [2].

Practice facilitation is underpinned by the understanding that primary care practices are frequently under-resourced, often lack the implementation experience and skills required, and of diverse contexts [3]. This complexity of primary care is what makes practice facilitation a successful implementation strategy, engaging a dedicated ‘facilitator’ (a role) to enable ‘facilitation’ (a process) of evidence-based innovations into practice. Facilitation, as an implementation strategy, can be defined as a process of enabling groups or teams to work together effectively to achieve a common goal [4]. Facilitation is a method that focuses not just on behaviour change, but also works toward changing the way individuals think and team culture [5, 6]. A systematic review identified that when facilitation is enacted in a way that tailors the implementation to the context and needs of the primary care setting, have significantly larger effect sizes [2]. Despite the success associated with practice facilitation, the level of complexity and tailoring involved can prove challenging for the facilitators. Practice facilitators are required to be mindful of each factor associated with facilitating innovations into practice not only during facilitation, but also must keep track of its status throughout facilitation (i.e. planning, guiding, and evaluation).

The profession of practice facilitation has grown substantially over the past decade with support from the Agency for Healthcare Research and Quality (AHRQ), emergence of multiple practice facilitator training programs, and an increasing interest in the research around knowledge translation [7]. Implementation science is an academic field that focuses on this research by developing and refining various approaches and theories to inform systematic implementation of innovations into healthcare systems [8]. These implementation theories, frameworks, and models can support practice facilitation by guiding how to predict, plan, and evaluate implementation efforts.

One such framework is the integrated-Promoting Action on Research Implementation in Health Services (i-PARIHS) framework [5], which highlights facilitation as the active ingredient for implementing innovations into practice. The i-PARIHS framework is the revised iteration of the original PARIHS framework and responds to several criticisms relating to the need for practical tools and case studies to help clinicians and researchers operationalise the framework [5, 6]. The i-PARIHS framework specifies core constructs and sub-constructs, which influence successful implementation, and aligns with practice facilitation, by being one of the first frameworks with an underlying philosophy that implementing research into healthcare practice is complex, unpredictable, and non-linear [5, 6].

Unfortunately, the existence of frameworks alone does not provide explicit guidance for facilitators. Like many frameworks, the original PARIHS framework received criticism for being difficult to operationalise, whereby resources and support are needed in order to effectively operationalise the constructs and how they relate to one another [9]. Therefore, in addition to the revised theory and clearly specified framework constructs, the developers of i-PARIHS provided several tools to operationalize i-PARIHS in practice, outlined in their published facilitation guide [5, 6]. This includes the *Facilitation Checklist* to support structured assessment of the framework constructs. See Table 1 for some examples of the questions from the Facilitation Checklist.

**Table 1:** Example of i-PARIHS Facilitation Checklist questions

i-PARIHS Facilitation Checklist questions	
Characteristics of the innovation	
Who is likely to be affected by the proposed innovation?	
What is the underlying evidence for the proposed innovation or change?	
Is the evidence packaged in an accessible and useable form (e.g. a clinical guideline, care pathway, or algorithm)?	
How much novelty does the evidence introduce?	
Does it offer advantages over the current way of doing things	
Is there potential to test out/pilot the introduction of the evidence/innovation on a small scale in the first instance?	
The recipients of the evidence/innovation	
Do individual members of the team want to apply the change?	
Do they perceive the proposed change as valuable and worthwhile?	

The Facilitation Checklist provides facilitators with various reflective questions to consider when working through and assessing the other i-PARIHS constructs (the innovation, recipients, and context). However, whilst these questions provide more operational support than the original PARIHS framework, it is important to evaluate whether the i-PARIHS Facilitation Checklist provides users with the appropriate support they need in order to successfully undertake facilitation.

Therefore, the current study took the i-PARIHS Facilitation Checklist to the North American Primary Care Research Group (NAPCRG)’s *2018 International Conference on Practice Facilitation*, Tampa, Florida. This conference brings together a large group of facilitators, researchers, and clinicians who are interested in practice facilitation. The focus of the 2018 conference was on ‘Building Capacity for Practice Facilitation’. Therefore, we felt this an excellent opportunity to conduct a workshop to build capacity for the attendees, whilst also evaluating the workshop in order to gain feedback around the i-PARIHS Facilitation Checklist as a resource, from those who work as facilitators ‘on the ground’.

This study is part of a larger body of work that seeks to adapt the i-PARIHS framework into a suite of practical and pragmatic resources (called the Mi-PARIHS Project—Mobilising Implementation of i-PARIHS). The current small-scale study explores how helpful and useful an adapted version of the i-PARIHS Facilitation Checklist is to practice facilitators in planning, guiding, and evaluating their implementation and facilitation endeavours. We hope to use this feedback to refine the tools and continuously engage with participants throughout tool development.

Specifically, our objectives are as follows:

1. How helpful do practice facilitators find the workshop format and its components, as a way of being introduced to an i-PARIHS tool and how it can be used?
2. Do practice facilitators find an i-PARIHS tool useful and if so, in what ways do they feel it could be most useful?
3. What characteristics do practice facilitators find most useful in an i-PARIHS tool?

## Methods

### Setting

We conducted a 1-h workshop at the North American Primary Care Research Group (NAPCRG)’s *2018 International Conference on Practice Facilitation*, Tampa, Florida. All of the conference attendees were practice facilitators or work within primary care/practice facilitation in some way. Therefore, it was an excellent opportunity to provide education and support to practice facilitators on implementation informed facilitation, whilst also providing the chance for us to gain feedback on what facilitators in practice need in regard to support with operationalising the i-PARIHS framework in. This study was approved by the Flinders University Social and Behavioural Research Ethics Committee (project number: 8223), and all participants provided informed consent.

### Workshop overview

Author ALK provided a plenary 1-h presentation on facilitation within the i-PARIHS framework. Following this, authors SCH and BK conducted a 1-h workshop focused on the i-PARIHS framework titled ‘Mi-PARIHS: Practical tools to make framework-informed implementation and facilitation easier’. This workshop presented on the background of implementation science and the i-PARIHS framework and provided an overview of a case example of how facilitators on an implementation team previously used i-PARIHS in a project (see [10] for detail on this project). The workshop then introduced attendees to the i-PARIHS Facilitation Checklist [5, 6]. In order for attendees to try using the Facilitation Checklist, a hypothetical facilitation scenario was provided which they could work through in small groups (3–5 people). However, participants were also free to work through the Facilitation Checklist whilst considering a project they were currently working on. To make the Facilitation Checklist useable in the context of a workshop, it was adapted from questions for reflection, to an itemised instrument (see Additional file 1). This allowed attendees to respond to each question with a rating between − 2 and +2. Negative responses (− 2 and − 1) indicated that the particular question was considered a barrier, a neutral response (0) indicated that the particular question was neutral, and positive responses (+1 and + 2) indicated that the particular question was considered an enabler. This rating scale was developed purely for the purpose of making the Facilitation Checklist interactive for participants and allow them to get a sense of how the questions are used to consider the constructs of the i-PARIHS framework and how they can inform facilitation strategies (through identifying enablers and barriers). To finalise the workshop, the authors’ pre-populated responses were provided as an example so we could demonstrate how using a rating scale to answer the Facilitation Checklist questions enables the generation of an associated visual representation (radar diagram; see Additional file 2) to assist with identifying the constructs that require more dedicated facilitation.

### Data collection

Following the workshop, all attendees were invited to participate in the research component by completing a paper-based survey to provide their feedback on using the i-PARIHS tool (see Additional file 3 for the survey). The survey included 17 questions which included basic demographics, quantitative questions relating to their experience of the workshop and feedback on the tool, and finally three open-ended questions to provide participants an opportunity to provide more detailed feedback. Participants were provided with an information sheet, and completion of the survey was indicative of consent to participate. Those who did not want to participate in the study were informed to not complete a survey.

### Data analysis

Workshop attendance was voluntary, so the number of participants that we could recruit for the evaluation survey was outside of our control. This resulted in a small sample size, and therefore, survey responses were analysed using descriptive statistics. The open-ended items were analysed qualitatively—authors SCH and BK independently reviewed the responses, conducted separate content analyses [11], and developed inductive codes for categories grounded in the data. The two authors (SCH and BK) then met to discuss and resolve discrepancies.

## Results

The following results are presented in four sections: (i) demographics of the participants, (ii) feedback on the *conference workshop*, (iii) feedback on the *use of the tool*, and (iv) feedback on the *characteristics of the tool*.

### Demographics

As attendance to the workshop was separate to the research component, the number of workshop attendees was not recorded. On estimate, out of approximately 30 workshop attendees, 10 participants completed the evaluation survey. Of these 10 participants, most identified as working within a facilitator role (80%) (Table 2).

**Table 2:** Summary of facilitation roles and experience

Survey question	Number	Percentage
Current professional role		
Full-time practice facilitator	4	40
Part-time practice facilitator*	4	40
Not currently a practice facilitator	2	20
Total	10	100
Facilitation experience**		
Expert facilitator	1	10
Experienced facilitator	6	60
Beginner or novice facilitator	2	20
N/A	1	10
Total	10	100

*The other aspects of their role varied from research coordinator (1), trainer and investigator (1), manager (1), and support consultant (1)

**Harvey and Kitson ([5, 6], pp. 80–81) outline three facilitator roles based on experience

The average years worked in their current role was 6 years (minimum 1 year, maximum 15 years). Harvey et al. [6] outlined a facilitation pathway from novice to experienced and expert facilitator, assuming different roles in the process of implementation. Table 2 outlines where the participants identified on this pathway.

### Workshop

The response to the overall experience of the conference workshop was positive with no negative responses (Table 3).

**Table 3:** Summary of overall experience of workshop

Overall experience	Number	Percentage
Very good	3	30
Good	4	40
Neither good nor bad	3	30
Total	10	100

As an introduction to the importance of implementation frameworks and information on the i-PARIHS framework, 40% of participants reported this as very valuable, 50% as valuable, and 10% as neither valuable nor not valuable.

The case example that provided an overview of how i-PARIHS can be used in a real project was largely found to be helpful, but responses to how helpful working through a hypothetical facilitation scenario with the i-PARIHS tool were more mixed (Table 4).

**Table 4:** Summary of how helpful participants found the case example and the facilitation scenario

Responses	Case example		Facilitation scenario	
	*N*	%	*N*	%
Very helpful	3	30	3	30
Helpful	4	40	3	30
Neither helpful or unhelpful	2	20	1	10
Unhelpful	1	10	2	20
Very unhelpful	0	0	1	10
Total	10	100	10	100

#### Qualitative responses

Qualitative responses help contextualize some of the quantitative responses. Participants mentioned there was not sufficient time during the workshop to fully comprehend the case study and work through the tool. Overall though, participants found the workshop important and useful, reporting that they learnt a lot about the i-PARIHS framework and the types of questions they should be asking. Further, they felt more equipped to be systematic in evaluating facilitation and how to use a tool in action. Some participants reported on the interesting nature of this work and encouraged the development of these tools that they find would be helpful in their everyday work.

### Use of the tool

Overall, participants responded positively to the idea of using the tool in their everyday role. Specifically, 50% of participants stated that the tool is likely something they would use, 20% said they would use it, 20% said not likely, and 10% said this question was not applicable. Table 5 outlines their responses regarding specifically using the tool for planning, monitoring, or evaluating their facilitation work.

**Table 5:** Summary of how helpful participants found the tool for planning, monitoring, and evaluation

Responses	Planning		Monitoring		Evaluating	
	*N*	%	*N*	%	*N*	%
Very helpful	3	30	1	10	2	20
Helpful	4	40	4	40	3	30
Neither helpful or unhelpful	1	10	3	30	4	40
Unhelpful	2	20	2	20	1	10
Very unhelpful	0	0	0	0	0	0
Total	10	100	10	100	10	100

### Characteristics of the tool

A unique aspect of the tool is its ability to generate a visual representation (radar diagram) of the three dimensions of i-PARIHS (innovation, recipient, context) based on the questions. Table 6 demonstrates how helpful the participants found these visual representations. See Additional file 2 for an example of these visual representations—radar diagrams.

**Table 6:** Summary of how helpful participants found the visual representations

Visual representations	Number	Percentage
Very helpful	2	20
Helpful	3	30
Neither helpful nor unhelpful	4	40
Unhelpful	1	10
Total	10	100

#### Qualitative responses

For the purpose of tool design (refinements and additional characteristics), the current study design collected a wide range of responses from participants that will increase the tool’s useability for practice facilitators. The specific suggestions include the following:

- An electronic tool that summarises and produces the visual representation
- Each stakeholder group needs own rating box/column
- Give the response options − 2 to +2 labels such as ‘strongly agree’, etc.
- Clarify questions to only address one concept at a time
- Comparing against authors’ scores was helpful
- Overlap between items—have facilitators narrow it down for what is meaningful
- Concerns that the tool is too subjective and user dependent to evaluate outcomes
- Simplify concepts and language
- Guidance on i-PARIHS domains/constructs prior to using the tool would be helpful

## Discussion

Practice facilitators are responsible for facilitating the implementation of clinical innovations into practice. Therefore, it is important for them to successfully implement, then also evaluate and demonstrate this success. Implementation frameworks enable this, but the existence of these frameworks alone does not provide sufficient support or guidance for practice facilitators. Therefore, the current study introduced practice facilitators to an implementation framework, i-PARIHS, and engaged them in a workshop to try out and use a practical tool based on the i-PARIHS Facilitation Checklist that they can use in their everyday practice.

Overall, the attendees at the conference were highly engaged and enthusiastic about the i-PARIHS framework and the refinement and development of practical tools and resources. Many of these attendees highlighted their limited understanding and use of implementation frameworks and emphasised how they found the i-PARIHS framework intuitively, very helpful. The survey results illustrate this enthusiasm and provide us with direction on the next steps needed for refinement and further development of the tool.

Specifically, we had three objectives. The first related to how practice facilitators found the workshop format and its components, as a way of being introduced to the i-PARIHS Facilitation Checklist. Participants had an overall positive experience with the workshop and found the introduction to implementation frameworks and the i-PARIHS framework valuable. This demonstrates that utilising conferences and interactive workshops are effective ways to educate practice facilitators on implementation science and introduce them to practical tools. Overall, this study supports the use of workshops as a way of introducing and spreading later iterations of the tool.

Our second objective focused on whether practice facilitators found the i-PARIHS Facilitation Checklist tool useful and in what ways. Half of the participants reported they would use the tool in their everyday practice which supports the rationale for further development and refinement of practical i-PARIHS tools. The positive response to the tool suggests that the Facilitation Checklist [5] is useful in operationalising the i-PARIHS framework and adapting it into a useable tool was a useful starting point. Specifically, participants saw the tool as most helpful in planning their facilitation and implementation efforts, followed by monitoring these efforts and then evaluating their overall success. This demonstrates how practice facilitators find support in planning most crucial, and therefore important that we focus efforts on developing a tool that can provide this support.

The final objective related to tool characteristics that practice facilitators find most useful. This helps us decide what refinements to this first version of a tool are necessary. Participants appreciated the ability of the tool to enable a visual representation (radar diagram) of the i-PARIHS dimensions, making it easy to determine which dimensions required the most facilitation. Through the qualitative feedback, participants encouraged the development of an electronic version of the tool which produces this visual representation, which supports its utility. Overall, the level of detail in which participants provided feedback on tool characteristics demonstrates their engagement and enthusiasm around its development.

As previous research identified, the original PARIHS framework received criticism for being difficult to operationalise [9] and the current study provides evidence that there remains demand and support for the development of i-PARIHS tools and resources to assist in using the i-PARIHS framework in practice. Taking what we have learned from meeting our three objectives, our planned next steps for i-PARIHS tool development are to (i) continue to utilize the workshop format for sharing and refining tools, (ii) allocate a considerable component of our development efforts to tools that assist practice facilitators with planning, (iii) focus on modes of tool provision that keep user-friendliness in mind (e.g. through making electronic versions available), and (iv) identify what the key questions are from the i-PARIHS facilitation checklist and translate the checklist from academic language into more practical and user-friendly language.

### Limitations

This study has several limitations. First, we had access to a limited number of participants for recruitment due to attendance at the workshop being completely optional and that the workshop was conducted at a conference that had concurrent sessions running. However, we felt it important to utilise this opportunity where a large number of practice facilitators were physically located at the one conference which resulted in greater success of recruitment compared with other means. Second, survey responses were not as rich as qualitative interviews, leaving some questions unanswered and detailed feedback limited. However, the time-efficiency associated with survey feedback that could be conducted at the time of the workshop outweighed the limitations. Finally, i-PARIHS is an implementation framework that can be, and is used, across multiple settings (primary care, acute care, community care, etc.). Therefore, more work is required to actively involve and seek feedback from facilitators who work across different settings, not just practice facilitators working in primary care, as we move forward with our larger Mi-PARIHS program of work.

## Conclusions

Despite a small sample size, this study was able to accomplish what we set out to do. Before embarking on a large program of work called the Mi-PARIHS Project where we will develop i-PARIHS tools and resources, we utilised this conference workshop opportunity, to gain some insight from those who are likely to use these tools and determine the need for their development, and what these users would like from i-PARIHS tools. This study has provided us with the reassurance that these tools will be used and will provide practical support for successful implementation.

Finally, this study serves as an example for other tool development endeavours. We have demonstrated how to effectively engage a convenient sample of end-users for feedback to direct and refine tool development, ensuring their feedback is utilised prior to a testing phase and is incorporated in the development stage. This study also demonstrates an effective way to disseminate and educate those who work in the field around the various theories and frameworks that support and inform their work. Overall, our approach could be tailored to other tool development endeavours’ needs and the lessons learned through our results in this study (e.g. in terms of user preference) are likely to be applicable to those other development endeavours as well.

## Supplementary information

**Additional file 1:.** Itemised instrument.

**Additional file 2:.** Visual representations—radar diagrams.

**Additional file 3:.** Paper-based survey.

## Data Availability

All quantitative data collected or analysed during this study are included in this published article. Raw qualitative data are not publicly available due to them containing information that could compromise participants’ privacy; derived data supporting the findings of this study are available from the corresponding author upon request.

## Abbreviations

AHRQ: Agency for Healthcare Research and Quality
i-PARIHS: Integrated Promoting Action on Research Implementation in Health Services
NAPCRG: North American Primary Care Research Group
PARIHS: Promoting Action on Research Implementation in Health Services
