# Supplementary material for: Interactive workshop to develop implementation framework (i-PARIHS) resources to support practice facilitation
Source: Implement Sci Commun. 2020 Jun 18;1:56. doi: 10.1186/s43058-020-00046-0 (PMC7427849; doi:10.1186/s43058-020-00046-0)
Supplement: Supplementary file 1 — Additional file 1:. Itemised instrument. [file 43058_2020_46_MOESM1_ESM.docx]

| **-2** | **-1** | **0** | **+1** | **+2** |
| --- | --- | --- | --- | --- |

**Response options:**

| Domain | Domain questions | Response |
| --- | --- | --- |
| INNOVATION | Is it derived from research, clinical consensus, patient view, local information/data – or a combination of these? |  |
|  | Is it viewed as rigorous and robust? |  |
|  | Is there a shared view about the evidence? |  |
|  | How well does it “fit” the local setting? |  |
|  | Is it likely to be accepted or contested by those people who have to implement it? |  |
|  | Is the evidence packaged in an accessible and useable form? (E.g. a clinical guideline, care pathway or algorithm)? |  |
|  | Will people be able to see easily and clearly what is proposed in terms of clinical practice and the process of patient care? |  |
|  | How much novelty does the evidence introduce? |  |
|  | Will it require significant changes in the process and/or systems of care delivery? |  |
|  | Will it present a challenge to people’s ways of thinking, mental models and relationships? |  |
|  | What are the implications of this in terms of the likely boundaries that will be encountered? |  |
|  | Will a knowledge transfer, translation or transformation strategy be required? |  |
|  | Does it offer advantages over the current way of doing things? |  |
|  | Is there potential to test out/pilot the introduction of the evidence/innovation on a small scale in the first instance? |  |

**Domain average score:**

| **-2** | **-1** | **0** | **+1** | **+2** |
| --- | --- | --- | --- | --- |

**Response options:**

| Domain | Domain questions | Response |
| --- | --- | --- |
| RECIPIENTS | **Individual level** | |
|  | Do individual members of the team want to apply the change in practice? |  |
|  | Do they perceive the proposed change as valuable and worthwhile? |  |
|  | Do they see a need to make the change? |  |
|  | Is the change consistent with their existing values and beliefs? |  |
|  | Are there local opinion leaders? Will they be supportive or obstructive? |  |
|  | Are individual members able to implement the proposed change? |  |
|  | Do they understand what the change entails? |  |
|  | Is it within their current level of knowledge and skills? |  |
|  | Will additional training and development be needed? |  |
|  | Do people understand the modifications that are needed and how to change and embed these? |  |
|  | Do individuals have the necessary authority to carry out the proposed changes? |  |
|  | Have key individuals whose support is needed been identified? Are they engaged in discussing/planning? |  |
|  | **Team level** | |
|  | At a collective level, does the team want to apply the change in practice? |  |
|  | Is the proposed change seen as valuable and worthwhile? |  |
|  | Do they see a need to make the change? |  |
|  | Is there a shared view or are there differences of opinion? |  |
|  | Is there existing data that can be used to highlight the potential for improvement? Or can you collect data for this purpose? |  |
|  | Are the team able to implement the proposed change? |  |
|  | Do they understand what the change entails? |  |
|  | Is it within their current level of knowledge and skills? |  |
|  | Will additional training and development be needed? |  |
|  | Does the team understand the modifications that are needed and how to change and embed these? |  |
|  | Does the team have the necessary authority to carry out the proposed changes? |  |
|  | Is there good inter-professional collaboration and teamwork? |  |
|  | Will support be needed to develop more effective collaboration and teamwork? |  |
|  | Are the potential barriers to implementation known? Are there strategies in place to address these? |  |
|  | Are the resources available to support the implementation process? |  |

**Domain average score:**

| **-2** | **-1** | **0** | **+1** | **+2** |
| --- | --- | --- | --- | --- |

**Response options:**

| Domain | Domain questions | Response |
| --- | --- | --- |
| INNER CONTEXT | **Local context** | |
|  | Are the formal and informal leaders likely to be supportive of the proposed change? |  |
|  | Are the formal and informal leaders helping to create a facilitative context through providing motivation and support, creating a vision and reinforcing the change process? |  |
|  | Is there a distributed and devolved style of manage? |  |
|  | Is there a culture that supports innovation and change? |  |
|  | Do staff feel actively involved in decisions that affect them? |  |
|  | Are staff trusted to introduce new ideas into practice? |  |
|  | Do staff and patients feel valued? |  |
|  | What is the past experience of introducing changes at a local level? |  |
|  | Are there mechanisms in place to support learning and evaluation and to embed the changes? |  |
|  | **Organisational context** | |
|  | Does the proposed change align with the strategic priorities for the organisation? |  |
|  | Has the support of key individuals and leaders within the organisation been sought and secured? |  |
|  | Is there a culture that supports innovation and change? |  |
|  | Is there a history of successful and sustained change within the organisation? |  |
|  | Does the organisation have systems and processes in place that support innovation and change? |  |
|  | Do the senior management team actively seek opportunities for improvement and encourage ideas and feedback from patients, the public and staff? |  |
|  | Are there mechanisms in place for embedding changes in routine practice? |  |
| OUTER CONTEXT | Does the proposed change align with the strategic priorities for the wider health system? |  |
|  | Are there incentives in the wider health system that reinforce the proposed change (e.g. pay for performance schemes, regulatory requirements, etc.)? |  |
|  | Are there inter-organisational networks (e.g. specialised clinical networks) that will be helpful in terms of supporting the proposed changes? |  |
|  | How much stability/instability is there in the wider health system? |  |
|  | Is this likely to influence the implementation project? |  |

**Domain average score:**
