# Supplementary figures and images for: Interactive workshop to develop implementation framework (i-PARIHS) resources to support practice facilitation
Source: Implement Sci Commun. 2020 Jun 18;1:56. doi: 10.1186/s43058-020-00046-0 (PMC7427849; doi:10.1186/s43058-020-00046-0)

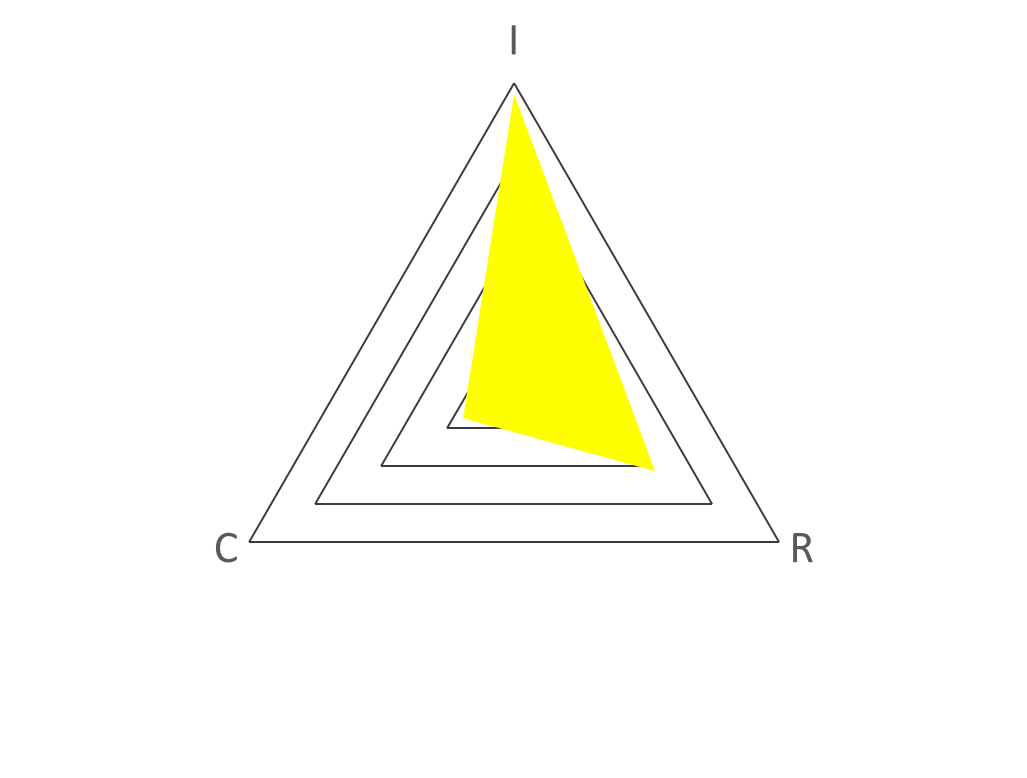


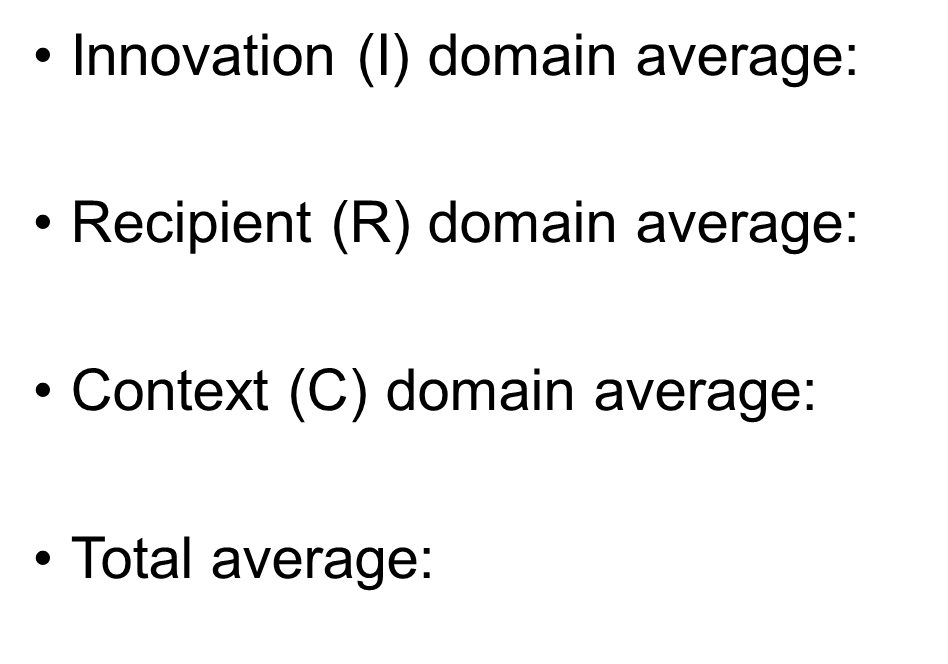


0.93

0.07

-0.62

0.13

Supplement: Supplementary file 2 — Additional file 2:. Visual representations—radar diagrams. [file 43058_2020_46_MOESM2_ESM.docx]
