# Supplementary material for: Interactive workshop to develop implementation framework (i-PARIHS) resources to support practice facilitation
Source: Implement Sci Commun. 2020 Jun 18;1:56. doi: 10.1186/s43058-020-00046-0 (PMC7427849; doi:10.1186/s43058-020-00046-0)
Supplement: Supplementary file 3 — Additional file 3:. Paper-based survey. [file 43058_2020_46_MOESM3_ESM.docx]

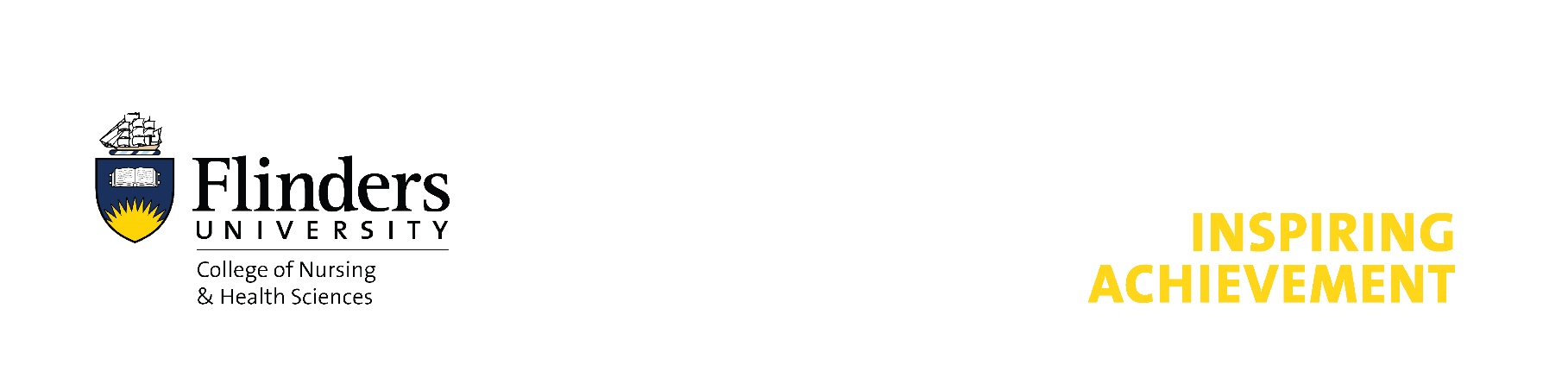
 ­­­­­

**Feedback Form**

**‘Mi-PARIHS: Practical tools to make framework-informed implementation and facilitation easier’**

1. What is your current professional role? _________________________________________
2. How many years have you worked in this role? ___________________________________
3. If you work as a facilitator, would you consider yourself a:

**Novice Experienced Expert Not applicable**

1. How would you rate the workshop overall: 1 (very poor) to 5 (very good)

**1 2 3 4 5**

1. How valuable did you find the information on the importance of implementation frameworks and i-PARIHS: 1 (not valuable) to 5 (very valuable)

**1 2 3 4 5**

1. How helpful did you find the case study example of how i-PARIHS can been used to assist in planning, guiding and evaluating evidence implementation: 1 (very unhelpful) to 5 (very helpful)

**1 2 3 4 5**

1. Do you think the Mi-PARIHS tool would be helpful in assisting with planning for facilitation: 1 (very unhelpful) to 5 (very helpful)

**1 2 3 4 5**

1. How helpful was the Mi-PARIHS tool when working through the case study example: 1 (very unhelpful) to 5 (very helpful)

**1 2 3 4 5**

1. Is the Mi-PARIHS tool something you would use?

**YES LIKELY** **NOT LIKELY NO**

1. How helpful was the Mi-PARIHS tool in monitoring the implementation progress in the case study example: 1 (very unhelpful) to 5 (very helpful)

**1 2 3 4 5**

1. How helpful were the visual representations (radar diagrams) of progress: 1 (very unhelpful) to 5 (very helpful)

**1 2 3 4 5**

1. How helpful do you think the Mi-PARIHS tool would be in evaluating implementation success: 1 (very unhelpful) to 5 (very helpful)

**1 2 3 4 5**

1. What were the most important things you learnt from this workshop?

____________________________________________________________________________________________________________________________________________________________________________________________________________________________________________________________________________________________________________________________

1. Do you have any thoughts on the characteristics or features of the Mi-PARIHS tool that would make it more useful/helpful for practice facilitators?

____________________________________________________________________________________________________________________________________________________________________________________________________________________________________________________________________________________________________________________________

1. Do you have any other comments or thoughts?

____________________________________________________________________________________________________________________________________________________________________________________________________________________________________________________________________________________________________________________________

1. We value your feedback and would therefore like to contact you in the future in regards to this project, would you be willing to participate further?

**YES NO**

1. If yes, please provide us with your email address: ________________________________

**Thank you for your feedback**
